# Organoids: The current status and biomedical applications

**DOI:** 10.1002/mco2.274

**Published:** 2023-05-17

**Authors:** Siqi Yang, Haijie Hu, Hengchung Kung, Ruiqi Zou, Yushi Dai, Yafei Hu, Tiantian Wang, Tianrun Lv, Jun Yu, Fuyu Li

**Affiliations:** ^1^ Division of Biliary Tract Surgery Department of General Surgery West China Hospital Sichuan University Chengdu Sichuan Province China; ^2^ Krieger School of Arts and Sciences Johns Hopkins University Baltimore Maryland USA; ^3^ Key Laboratory of Rehabilitation Medicine in Sichuan Province West China Hospital Sichuan University Chengdu Sichuan China; ^4^ Departments of Medicine Johns Hopkins University School of Medicine Baltimore Maryland USA; ^5^ Departments of Oncology Johns Hopkins University School of Medicine Baltimore Maryland USA

**Keywords:** 3D culture, drug screening, precision medicine, stem cells, tumoroid

## Abstract

Organoids are three‐dimensional (3D) miniaturized versions of organs or tissues that are derived from cells with stem potential and can self‐organize and differentiate into 3D cell masses, recapitulating the morphology and functions of their in vivo counterparts. Organoid culture is an emerging 3D culture technology, and organoids derived from various organs and tissues, such as the brain, lung, heart, liver, and kidney, have been generated. Compared with traditional bidimensional culture, organoid culture systems have the unique advantage of conserving parental gene expression and mutation characteristics, as well as long‐term maintenance of the function and biological characteristics of the parental cells in vitro. All these features of organoids open up new opportunities for drug discovery, large‐scale drug screening, and precision medicine. Another major application of organoids is disease modeling, and especially various hereditary diseases that are difficult to model in vitro have been modeled with organoids by combining genome editing technologies. Herein, we introduce the development and current advances in the organoid technology field. We focus on the applications of organoids in basic biology and clinical research, and also highlight their limitations and future perspectives. We hope that this review can provide a valuable reference for the developments and applications of organoids.

## Graphical abstract

Organoids can be generated by inducing and culturing pluripotent stem cells (iPSCs), embryonic stem cells (ESCs), adult stem cells (ASCs), and tumor cells from healthy donors or patients. The potential biomedical applications for organoids include disease modeling, drug screening, toxicity assays, personalized medicine, biobank, biomarker discovery, and regenerative medicine.

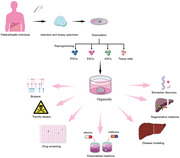

## 1 INTRODUCTION

Research on organoids can be considered to be traced back as far as 1907, when Wilson et al. cultivated mechanically dissociated sponge cells to form functional organisms under in vitro conditions.^1^ In the decades since, organoid research has been carried out mainly through the isolation and reorganization of cells. In 1975, a study incubated primary human keratinocytes and 3T3 fibroblasts to generate stratified squamous epithelial colonies similar to those of the human epidermis.^2^ By the 1980s, the first pluripotent stem cells (PSCs) were isolated from mouse embryos and obtained by humans, and mesenchymal stem cells (MSCs), human embryonic stem cells (ESCs), and induced PSCs (iPSCs) were successively discovered.^3^ The development and advances of stem cell technology shed new light on the field of organoids. In 2009, intestinal adult stem cells (ASCs) were cultured in vitro to form small intestinal organoids with a crypt‐villi structure,^4^ which is a landmark event in the organoid field, demonstrating the potential of stem cells to differentiate into spatial structures similar to organs in vivo. Since then, organoid culture techniques have flourished and organoids derived from various organs have been established, such as the brain,^5^, ^6^ retinal,^7^, ^8^ lung,^9^, ^10^ stomach,^11^, ^12^ liver,^13^, ^14^ bile duct,^15^, ^16^ pancreas,^17^, ^18^ and kidney.^19^, ^20^

The rapid development of three‐dimensional (3D) culture technologies exerts crucial functions in enabling organoid culture. Cells in the body reside in complex internal environments, and they are affected by multiple signals and interactions to establish, maintain, and regulate cellular phenotypes and specific functions. Cells cultured in two dimensions (2D) failed to recapitulate the normal cell morphology and interactions in vivo. Isolated tissue cells cultured in a 2D system gradually lose their morphology and become flattened, abnormally divided, and influence the cellular differentiated phenotype.^21^, ^22^ 2D attachment tends to cause cells to lose their original shape and hierarchical structure and affect cell–cell and cell–extracellular signal transduction and interactions, resulting in cells that do not properly reproduce the cellular functions and behaviors present in tissues or organs.^23^ 2D tumor cell lines gradually lose their heterogeneity during long‐term culture. Meanwhile, cross‐contamination easily occurs in long‐term subculture. It has been confirmed that the genomics and metabolomics of cell lines are significantly different from those of the original tumor after long‐term passage.^24^ The 3D culture system can mimic the physicochemical microenvironments and intercellular and cell–ECM interactions in which cells live in vivo.^25^ Cells cultured in a 3D system can better represent the complex structure and specific cellular functions of cells, which have a high degree of fit with the parent and maintain the genetic stability and chromatin heterogeneity of the parent as well. These cells can expand rapidly in 1−2 weeks and be stably subcultured and cryopreserved.^26^

Organoids can be defined as cells with stem cell potential that are incubated under 3D culture systems to aggregate by adhesion, self‐organize, and differentiate into 3D cell masses with the corresponding organ tissue morphology.^27^ Organoids have a high degree of similarity to their parental cells that replicate and simulate their unique biological characteristics.^28^ Additionally, organoids are able to self‐renew and self‐organize, contain various cell types, perform some specific functions, and form spatial structures similar to those of in vivo organs. Therefore, organoids are valuable models for studying the occurrence, development, and progression of diseases. Tumor organoids can be constructed by preoperative biopsy or postoperative tumor resection, which plays an important role in individualized drug sensitivity prediction. Thus, organoid models provide better options for drug screening and individualized drug therapy.^29^, ^30^, ^31^, ^32^, ^33^ The potential of organoids to broaden fundamental research by complementing current model systems is becoming more generally recognized.^34^

Organoid technologies pave the way for the development of new models that more closely resemble realistic physiological and pathophysiological states. In this review, we introduced the development and current advances in the field of organoids and described the main cell sources and culture methods for organoids. We focused on the application of organoids in basic biology and clinical research, such as disease modeling and precision treatment, and also highlighted their limitations and future perspectives. We hope that this review can provide a valuable reference for the application of organoid technologies.

## 2 CELL SOURCES OF ORGANOID

**FIGURE 1:**
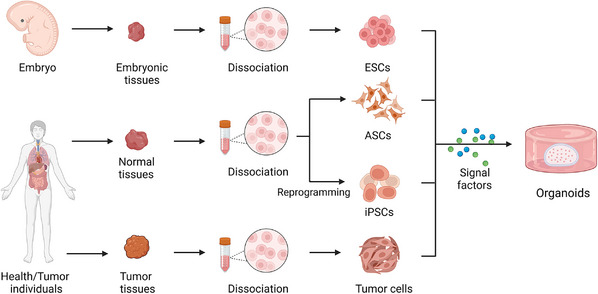
Strategies for formation of organoids in vitro. The cell sources for establishing organoids include embryonic stem cells (ESCs), adult stem cells (ASCs), induced pluripotent stem cells (iPSCs), and tumor cells. Resection and/or biopsy specimens from health/ patient individuals are dissociated into single cell to form organoids by incubating with various signal factors (Created with BioRender.com).

Stem cells are primitive, undifferentiated cells that are able to differentiate into many different and specialized cell types. Due to their potential for self‐renewal and multidirectional differentiation, organoids can be established from stem cells, including ESCs, iPSCs, and ASCs^4^, ^35^ (Figure 1). The process by which stem cells develop into organoids is similar to the way that organs acquire unique organization, mainly involving self‐organization.^36^

The following considerations need to be taken into account when cultivating organoids. First, owing to the 3D structure of organoids, the 3D culture environment should be provided, which can be achieved by embedding the organoids in a matrix,^37^ or by applying the air–liquid interface (ALI) culture technique.^38^ Second, establishing the appropriate regional identity is a key point in the organoid culture process, which requires the correct regulation of development and differentiation‐related signaling pathways. Finally, different types of organoids require different nutrients to develop and differentiate into the terminal stage. As a result, the medium needs to be configured according to the appropriate protocol.

### 2.1 PSC‐derived organoids

**FIGURE 2:**
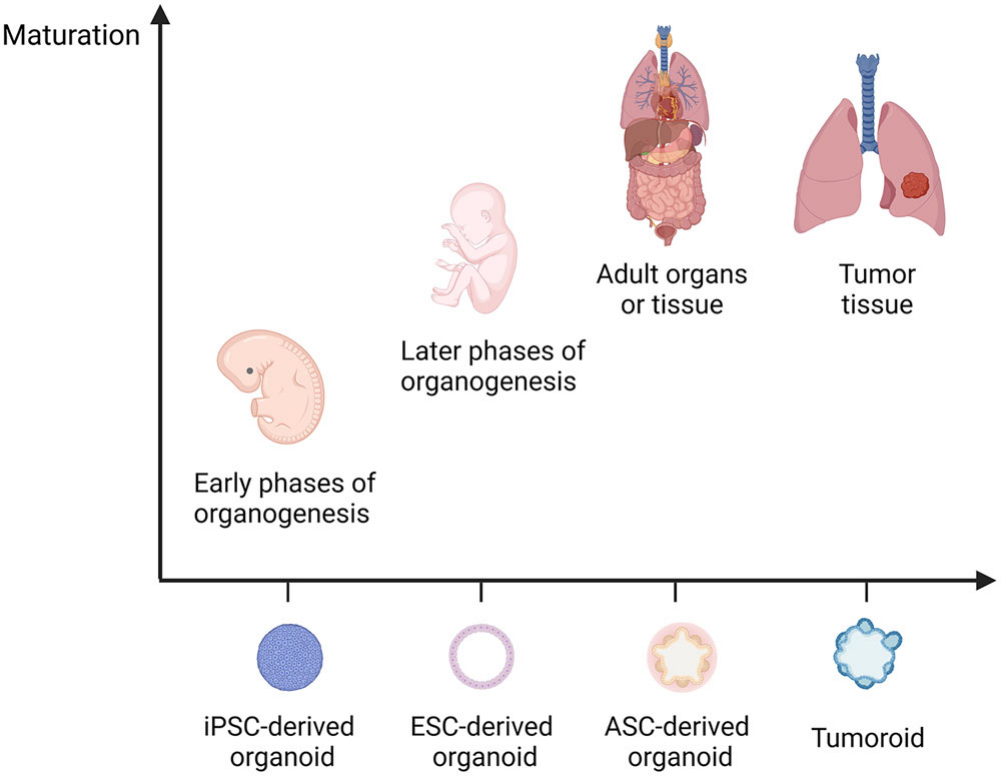
The maturation of different cell‐derived organoids. The iPSC‐derived organoids present low maturation, which are similar to the embryonic or early fetal tissues and can be used in studying organogenesis. The ESC‐derived organoids have higher maturation than iPSC‐derived organoids and are applied to model later organogenesis stage. The ASC‐derived organoids are more closely resembling adult tissue. Tumor cell‐derived organoids is a special case due to they represent adult tumor features (Created with BioRender.com).

Since the discovery of PSCs, researchers have generated a wide range of differentiated cell types from stem cells based on theories of developmental biology.^39^, ^40^ iPSCs are produced by reprogramming PSCs (such as by integrating transcription factors into the target cells), and ESCs are derived from cell masses within the blastocyst, both of which have the capacity for multidirectional differentiation.^41^, ^42^ The establishment of PSC‐derived organoids depends on the directed differentiation of PSCs. This process requires the formation of specific germ layers (endoderm, mesoderm, or ectoderm), followed by incubating with specific growth factors, signaling molecules, and cytokines to induce cell‐directed differentiation and maturation. PSC‐derived organoids contain a richer cellular fraction, including mesenchymal, epithelial, and endothelial cells.^43^, ^44^ iPSC‐derived organoids were applied in modeling various human diseases in vitro.^45^, ^46^, ^47^, ^48^, ^49^ However, when PSC‐derived organoids reach a certain lifespan, they lose their ability to proliferate and fail to develop to a fully mature state.^50^, ^51^ In addition, the limitations of current technologies cause the lack of important interactions between cells in PSC‐derived organoids with other codeveloping cells.^52^ Therefore, PSC‐derived organoids are generally naïve, resembling fetal tissues, which can be considered as excellent models for organogenesis research.^53^ ESC‐derived organoids are more mature than iPSC‐derived organoids, which can be used as novel models for studying later phases of organogenesis.^54^, ^55^ However, ESCs need to be obtained from the embryo and there are many ethical issues involved (Figure 2).

### 2.2 ASC‐derived organoids

ASCs (with the exception of skin stem cells) are thought to be incapable of significant in vitro expansion. The team of Clevers applied 3D technologies to culture the leucine‐rich repeat‐containing G protein‐coupled receptor 5 positive (Lgr5+) intestinal mouse ASCs to establish intestinal organoids by adding various stem cell niche factors into the medium and manually modulating signaling pathways. This study indicated that ASCs could expand in vivo in the presence of stem cell niche factors. The construction of ASC‐derived organoids requires identifying proper ASC types. WNT pathway activation is one of the main drivers of organoid formation in epithelial ASCs, and Lgr5 was positive in almost all epithelial ASCs that could be used for organoid culture. The culture protocol for ASC‐derived organoids is simpler, shorter, and more mature, and they more closely resemble adult tissue.^56^, ^57^ Therefore, they can be better used in regenerative medicine and disease modeling because their cell sources are abundant.^58^, ^59^, ^60^ However, ASC‐derived organoids have a single cellular component, mainly epithelial,^61^ and prior knowledge of the medium for culturing ASC‐derived organoids from different tissues is also a limitation for ASC‐derived organoids.

### 2.3 Tumor cell‐derived organoids

Based on the culture protocol for ASC‐derived organoids, organoids can be generated from isolated tissues. Isolated tumor tissues after digestion and dissociation can be used to grow organoids with 3D culture conditions, and these organoids are called tumoroids. Tumoroids maintain and preserve the histological structure, molecular genetic characteristics, and heterogeneity of the original tumor, which can represent the features of the patient's tumor in some degree.^62^, ^63^ Tumoroids can be used as good preclinical models for basic and clinical research related to tumors.^64^, ^65^, ^66^, ^67^, ^68^, ^69^, ^70^, ^71^, ^72^ Tumor liquid biopsy samples or tumor cells collected by the Pap brush method have been considered as starting material for organoid cultures.^73^, ^74^, ^75^, ^76^ De Angelis et al.^77^ constructed the mouse xenograft model by using colorectal circulating tumor cells to promote these tumor cells proliferation in order to generate sufficient materials to form tumoroids. In addition, whether by surgical resection, puncture biopsy, or samples collected by Pap brush, the isolated tumor cells usually included normal cells and/or blood cells (especially red blood cells), which might influence tumoroid growth.^77^ A possible solution to this problem is to reduce or delete some factors necessary for the establishment of normal cell‐derived organoids, as tumor cells reduce their dependence on them. To eliminate contamination of red blood cells, lysis is one method that can be adopted.^78^

### 2.4 Multilineages organoids

To represent the niche of cells in an organism, the interactions between various regions and different cell populations in physiology and pathophysiology should be recapitulated as closely as possible. For example, the interactions between different brain regions contribute to the formation of certain brain regions.^79^ Mesenchymal and endothelial cells have been found to exert indispensable functions in the development of the liver and are able to manipulate the behavior of hepatic progenitors.^80^ Assembloids, considered as the next generation of organoids, can be established by coculturing the multiple‐type cells or combinations of organoids with different cell lineages from other tissues or organs.^79^, ^81^, ^82^ Compared with single‐type cell‐derived organoids, assembloids can better reproduce interactions among different subregions or various cell lineages in organs.

Several studies fused cerebral organoids from different brain regions to capture the interactions between different brain regions in the process of brain development, including long‐distance projections and interneuron migration.^83^, ^84^, ^85^ A recent study by Andersen et al.^86^ formed cortical, hindbrain/cervical spinal cord, and skeletal muscle organoids, and fused the three organoids to generate cortico‐motor assembloids, which can represent the cortico‐motor pathway functions (the cortical control of muscle contraction) in vitro long term. Similarly, Ogawa et al.^87^ developed a novel cancer model of gliomas that was established by the artificial combination of brain organoids and glioblastoma cells, which can be used to study tumorigenesis and metastasis mechanisms.

Research based on the assembloid has also been extended to the peripheral nervous system. It has been reported that iPSCs can differentiate into spinal cord neurons and skeletal muscle cells simultaneously, and these cells can self‐organize to form hybrid neuromuscular organoids in vitro.^88^ Intestinal organoids cocultured with neural crest cells were confirmed to model the development of the enteric nervous system and motility disorders of the intestinal tract.^89^

Assembloids resembling other tissues or organs have been reported by many studies. Takebe et al.^90^, ^91^, ^92^ incubated iPSCs or human embryonic cells with human umbilical vein endothelial cells and human MSCs to generate 3D vascular and functional organ buds. Bergmann et al.^93^ generated blood–brain barrier (BBB) organoids via coculturing human primary brain endothelial cells, astrocytes, and pericyte. The anterior and posterior gut spheroids derived from iPSCs were cocultured under 3D conditions to generate assembloids that could accurately mimic the continuous and dynamic process of human hepatobiliary and pancreatic organogenesis in vitro.^94^ Similarly, Ng et al.^95^ formed cardio‐pulmonary assembloids by inducing iPSCs to differentiate cardiac and lung epithelial cells simultaneously, which is a promising platform for studying the interactions between the human heart and lung and tissue boundary formation during embryonic development. The teams of Chan et al. and Takebe et al. established multicellular hepato‐biliary organoids from iPSCs, and these organoids have the potential to model complex liver diseases such as nonalcoholic fatty liver disease (NAFLD).^96^, ^97^ In addition, Tanimizu et al.^98^ generated functional hepatobiliary organoids by combining EpCAM+ cholangiocytes and small hepatocytes, and found that a connection between hepatocytes and cholangiocytes was formed in the organoids.

## 3 CULTURE APPROACHES OF ORGANOIDS

### 3.1 Submerged culture

Submerged culture, developed by the group of Clevers, is the most widely used culture method for organoids.^37^ In this protocol, cells or cell clusters are embedded in extracellular matrix (ECM) gels, then the mixture of cells and matrix gels is placed in a culture dish to form a dome, and medium is added to submerge the dome. ECM gels play the role of structural support and offering ECM signals, and common ECM gels include basement membrane extract, Matrigel, and Geltrex. The main components of the medium for the submerged culture include adDMEM/F12, penicillin/streptomycin, GlutaMAX, HEPES, B27, epidermal growth factor (EGF), FGF2, FGF10, Wnt3A, Noggin, R‐spodin‐1, Prostaglandin E2, N‐Acetylcysteine, Nicotinamide, Y27632, A‐8301, and SB202190.^37^, ^99^ The medium composition varies when forming organoids from different tissues, and which specific factors need to be added or removed usually depends on the requirements of the tissue from which they originate for the corresponding signal or hormone.^100^

### 3.2 ALI culture

The 3D culture conditions for organoids can be achieved using ALI technology that was first proposed by Ootani et al.^101^ for the establishment of pancreatic and gastrointestinal tract organoids. The mechanically separated tissue fragments were homogeneously embedded in collagen gel, the mixture was laid flatly in an inner culture dish with a porous membrane underneath, and the top of the mixture was exposed to air. The medium was added in an outer culture dish. In this system, nutrients and growth substances are transferred by diffusion from the medium in the outer dish into the inner dish to meet the requirements of organoids.^102^, ^103^ Due to direct exposure to oxygen, ALI cultures have a higher oxygen supply than the submerged culture method. Kuo's group later established a variety of patient‐derived tumoroids using the ALI method, including some rare tumor types such as bile duct ampullary adenocarcinoma.^38^ The ALI culture system, combining two culture dishes, can support the growth of organoids and stromal cells simultaneously. It is worth noting that organoids formed with this approach are able to preserve the functional tumor‐infiltrating lymphocytes (TILs), which can represent the complex tumor immune environment.^38^, ^104^

### 3.3 Organoids‐on‐a‐chip

Organ‐on‐a‐chip is a microfluidic cell culture device that allows accurate control of the biophysical and biochemical environment for cell growth and simulates both cellular and microenvironmental conditions, as well as inter‐tissue and multiorgan interactions.^105^ A variety of organ‐on‐a‐chips have been created to simulate corresponding organs in vitro, which are used for disease modeling and to study the function of related organs.^106^, ^107^, ^108^

Although organ‐on‐a‐chip has fundamental differences from organoids, the organoids‐on‐a‐chip generated from the combination of organoid technologies and organ‐on‐a‐chip can compensate for the shortcomings of the two technologies, and thus better serve as more effective preclinical models for simulating key features of target organ tissues. Cells in organoids‐on‐a‐chip are randomly and spontaneously self‐organized into 3D structures, which differs from the carefully designed organ‐on‐a‐chip.^109^ Currently, a number of studies have established organoids‐on‐a‐chip of the brain, heart, gastrointestinal tract, liver, and pancreas.^110^, ^111^, ^112^, ^113^, ^114^, ^115^, ^116^ Cho et al.^110^ developed a brain organoids‐on‐a‐chip based on PDMS chips, and this system could increase the oxygen supply and promote nutrient/waste exchange to reduce the organoid cell death. Notably, this culture system could form mature brain organoids and be used to monitor the development of the overall human brain. A recent study has shown that the organoid‐on‐a‐chip system can create a hypoxic gradient in the lumen of small intestinal organoids and maintain the intestinal microbial barrier.^117^

In addition to the above culture methods, 3D systems for organoids can be prepared by the hanging drop and rotational culture methods. The hanging‐drop approach applied gravity and surface tension to hang the mixture of cells and droplets of specific medium from a plate.^118^ The rotational culture method can prevent cells from settling and improve the uptake of nutrients and oxygen by constantly rotating or stirring the cells, which has been used in the formation of brain and retinal organoids.^119^, ^120^ Jacob et al.^121^ generated patient‐derived glioblastoma organoids that preserve the histological and genetic features and part of the microvasculature and immune microenvironment.

## 4 BIOMEDICAL APPLICATIONS OF ORGANOIDS

**FIGURE 3:**
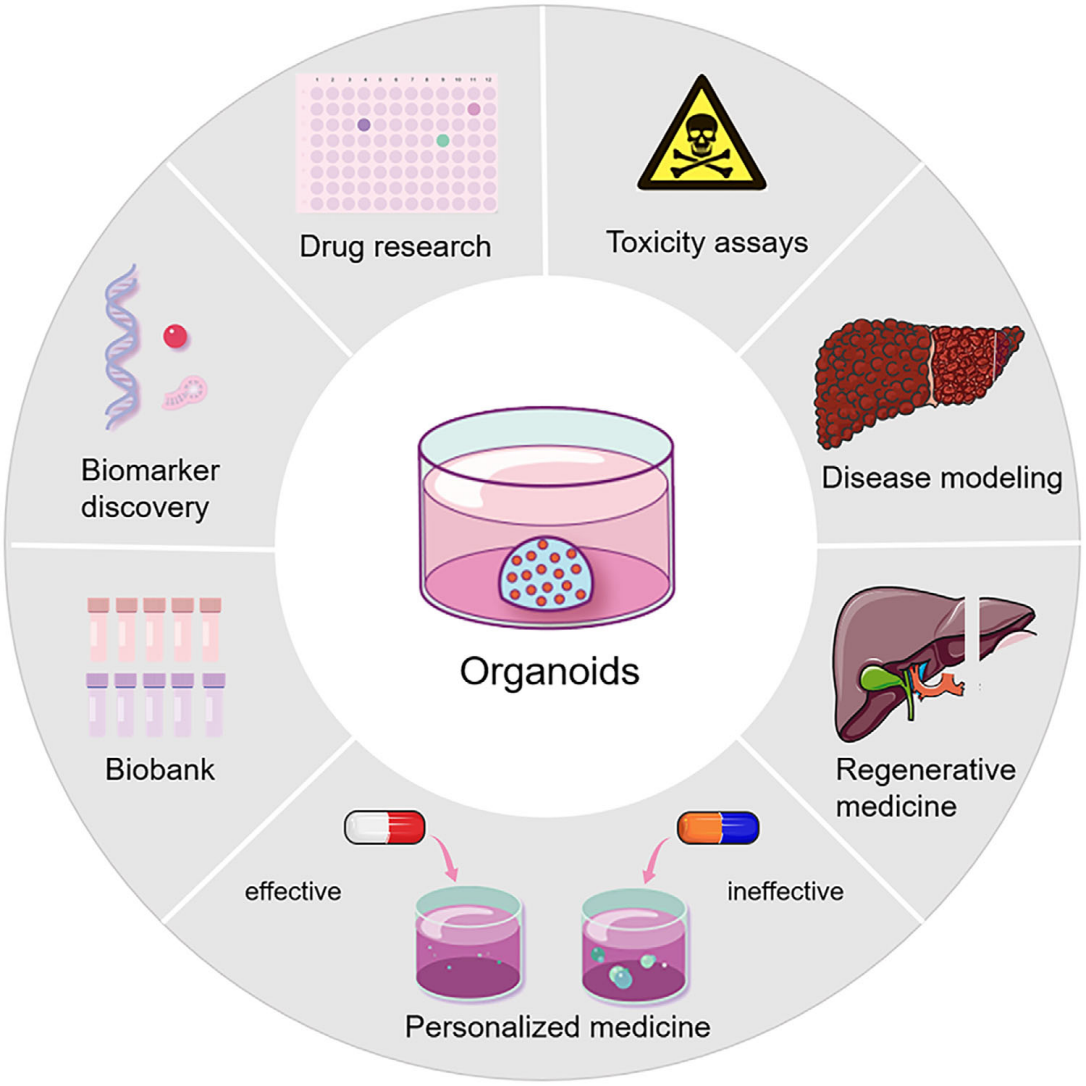
Biomedical applications of organoids. Organoids can be used as disease models to understand the mechanisms and physiopathology of human hepatobiliary diseases. Organoids are ideal models for drug screening and toxicity assays. Patient‐derived organoids can be used to predict patient‐specific responses to drugs and personalized treatment. Additionally, cryopreservation of organoids makes the establishment of biobank possible. Other biomedical applications of organoids include biomarker discovery and regenerative medicine.

The rapid development of hepatobiliary organoid technology has provided better options for studying cell development, tissue maintenance, and pathogenesis of the hepatobiliary system under physiological or pathological conditions closely resembling natural conditions. Organoids have the following significant advantages. First, organoids are human‐derived and can recapitulate human physiological systems. The second is high efficiency. ASC‐ or PSC‐derived organoids are quick and relatively easy to establish. Third, organoids show stability in all aspects during large‐scale genomic screening or drug screening. The last is individualization. Organoids can be generated from individual tissues or cells, contributing to the realization of precision diagnosis and treatment. These advantages endow hepatobiliary organoids with a wide range of biomedical applications (Figure 3).

### 4.1 Disease modeling

#### 4.1.1 Genetic diseases

Organoids can mimic the development of organs in vitro, which can be used to model and study the mechanisms of organ‐specific genetic diseases. Liver organoids were generated from patients with α1‐antitrypsin deficiency, and the A1AT protein was found to aggregate in organoid cells, which could represent the key pathological and pathophysiological features of α1‐antitrypsin deficiency.^122^, ^123^ Similarly, other hepatobiliary hereditary diseases, including Alagille syndrome, Wilson's disease, and Wolman disease, have been modeled with hepatobiliary organoids.^123^, ^124^, ^125^, ^126^, ^127^ The islet organoids have been unitized to model insulin secretion‐related genetic disorders such as Wolfram syndrome and congenital hyperinsulinism.^128^, ^129^

Cystic fibrosis (CF) is a monogenic hereditary disease, and the main clinical manifestations are dysfunction of the lungs, pancreas, liver, intestines, and reproductive system.^130^ CF is caused by mutations in the CF transmembrane conductance regulator (CFTR) gene, and over 360 variants have been identified as pathogenic mutations.^130^, ^131^ CFTR, belonging to the adenosine triphosphate (ATP) binding cassette family, is a cell‐surface chloride transporter, and mutations in CFTR impair epithelial cell function, leading to the occurrence of clinical symptoms. CF organoid models of the airway, intestine, hepatobiliary system, and pancreas from humans or mice have been established.^132^, ^133^, ^134^, ^135^, ^136^ Forskolin is a CFTR activator and has the ability to lead to intestinal organoid swelling. Forskolin‐induced swelling (FIS) of patient‐derived intestinal organoids has been shown to predict the response to treatment in CF patients.^137^ A recent study indicated that the degree of patient‐derived rectal organoid swelling induced by forskolin was associated with the severity of clinical symptoms in CF patients.^134^ Organoids can be used as a patient‐specific model for CF disease to study the molecular abnormalities associated with various CFTR mutations and develop new medicine strategies. Li et al.^138^ established iPSC‐derived organoid models of autosomal dominant polycystic kidney disease. Based on the model, the authors proposed that absorptive pathways, especially glucose absorption, play an important role in the formation of cysts. More recently, Kim et al.^139^ edited iPSC‐derived kidney organoids via CRISPR‐Cas9 technology to generate GLA‐mutant Fabry nephropathy models, whose applications ranged from studying pathophysiology to exploring novel therapeutic regimens in Fabry nephropathy. Cardiac organoids derived from HAND1 or NKX2‐5 knockout iPSC lines were prepared to model hypoplastic left heart syndrome.^140^ Pregestational diabetes‐induced congenital heart defects were also modeled by human heart organoids.^141^ Genetic diseases of organs originating from the neuroectoderm can be modeled by organoids as well. Retinal organoids have been used as powerful tools to model and investigate the mechanisms of inherited retinal diseases such as retinitis pigmentosa and Leber's congenital amaurosis.^142^, ^143^, ^144^, ^145^ Primary microcephaly is caused by single mutations or duplications of genes such as CDK5RAP2,^146^ whose organoid model was developed 10 years ago.^147^ In recent years, organoids have been established for different genetic mutations causing microcephaly.^148^, ^149^ Specific brain organoids of syndromic neurodevelopmental disorders, such as Rett syndrome,^150^ Tuberous sclerosis complex,^151^, ^152^ fragile X syndrome,^153^ Down syndrome,^154^ and Angelman syndrome,^155^ have been generated. The advent and rapid development of organoid technologies has provided important insights into the etiology, pathogenesis, and discovery of potential treatment alternatives for genetic disorders. The combination of gene‐editing technologies (such as CRISPR‐Cas9) and organoid models provides better options for studying genetic diseases that are complex and difficult to model in vitro.

#### 4.1.2 Infectious diseases

Owing to the fact that infectious agents usually infect specific species or cell types, human models are superior to animal models when investigating the pathogenetic mechanisms and development of these diseases. As described above, microcephaly is a developmental brain disorder. Recent studies revealed the associations between microcephaly and Zika virus (ZIKV) infection^156^, ^157^ and found that several compounds could reduce the cell death induced by ZIKV.^158^ Technical advances in 3D culture and organoids have made it possible to culture certain viruses in vitro. For instance, the culture system of human norovirus failed to be established for decades. Ettayebi et al.^159^ first succeeded in culturing human norovirus in vitro with epithelial monolayers derived from human intestinal organoids, and they optimized the culture system to allow the cultivation of multiple strains.^160^ Culture systems for influenza virus, human rotavirus, and BK virus have been established with organoids.^161^, ^162^, ^163^

Despite advancements in therapy, liver disorders caused by viral infections continue to be a national health concern in China. When hepatitis viruses invade and attack hepatocytes, the immune response is activated to kill viruses as well as virus‐infected hepatocytes, causing degeneration and necrosis of liver parenchymal cells with reactive stromal tissue hyperplasia, leading to the accumulation of collagen fibers in the liver. The extensive liver fibrosis and hepatocyte necrosis lead to the occurrence of liver remodeling and the formation of false lobules, which ultimately bring about cirrhosis and even liver failure.^164^, ^165^ Additionally, viral hepatitis and liver cirrhosis are also risk factors for hepatocellular carcinoma (HCC).^166^ Human liver organoids have emerged as advanced tools for studying hepatitis virus infection and several laboratories have generated liver organoids infected by hepatitis B, C, and E viruses.^167^, ^168^, ^169^, ^170^, ^171^ Recent research indicated that when liver organoids derived from healthy donors were cocultured with the recombinant virus or the serum of patients with HBVs, the organoids were infected, and the virus proliferated actively.^172^

Severe acute respiratory syndrome coronavirus 2 (SARS‐CoV‐2) is the pathogen of coronavirus disease 2019 (COVID‐19), which usually causes moderate respiratory disease such as fever and cough, and even acute respiratory syndrome.^173^ Alveolar lung airway and bronchial organoids have been generated and used to mimic SARS‐CoV‐2 infection in vivo, investigate COVID‐19‐related pathophysiology, and pathology and develop novel therapeutics.^10^, ^174^, ^175^ Additionally, COVID‐19 also leads to extrapulmonary disease, including gastrointestinal and neurological symptoms, and liver and kidney injury, and corresponding organoids have been applied in SARS‐CoV‐2‐related research.^174^, ^176^, ^177^, ^178^, ^179^

The applications of organoid models in infection biology are not limited to virus research but can also be used to study bacteria and protozoan parasites. Cryptosporidium infected human small intestinal and lung organoids have been established.^180^ Similarly, relationships between the BBB and *Plasmodium falciparum* and Lyme neuroborreliosis were explored via BBB organoids.^181^, ^182^ Bartfeld et al.^183^ successfully established *Helicobacter pylori* (Hp)‐infected human gastric organoids, and inflammatory responses were observed in these infected organoid cells. The adhesion of Hp depends on the high affinity between the TlpB receptor and urea secreted by gastric cells, and Hp is likely to attach to highly differentiated pit cells with a larger volume.^184^ Furthermore, the CagA factor derived from Hp was transferred into organoid cells, leading to activation of certain pathways and increased cell proliferation.^50^, ^184^ A recent study demonstrated that the Hp infection could activate NF‐κB signaling pathway and upregulate RAS protein activator like 2 (RASAL2) expression to promote proliferation of gastric tumor cells. Downregulation of RASAL2 could significantly inhibit the growth of gastric tumoroids.^185^ Murine gallbladder organoids with Salmonella Typhi infection contributed to malignant transformation (TP53 mutations and c‐MYC amplification) and activating AKT and MAPK signal pathways, which promoted abnormal cellular proliferation and transformation to tumors.^186^

To sum up, the 3D organoid models of infection biology can reflect the correlation and interaction between pathogenic microorganisms and host cells, providing crucial preclinical models for mechanistic exploration, treatment, and drug development for infectious diseases. Additionally, the establishment of a coculture system of organoids and oncogenic pathogens provides an advanced platform to further study the mechanism of tumor formation promoted by biological pathogens.

#### 4.1.3 Metabolic diseases

Currently, metabolic diseases pose a serious risk to health worldwide. However, the absence of proper models limits the exploration of underlying mechanisms and potential therapeutics. Organoid culture technologies shed new light on the research in this filed.

Obesity is one of the common metabolic diseases that is characterized by an increase in adipose tissue, increasing the risk of type 2 diabetes and NAFLD.^187^ Adipose organoids have been utilized to study fat‐related metabolism and model obesity.^188^, ^189^ Recent studies reported the formation of adipose organoids with immune cells or vascular systems, which could replicate the signal communication between adipocytes and other cells in vivo.^190^, ^191^

NAFLD is a group of disorders correlated with metabolic dysfunction, including nonalcoholic fatty liver and nonalcoholic steatohepatitis (NASH), which may eventually lead to liver cirrhosis and HCC. Additionally, it has been confirmed that NAFLD is associated with type 2 diabetes and can increase cardiovascular risk.^192^, ^193^ Ouchi et al.^127^ generated iPSC‐derived liver organoids encompassing hepatocyte‐, stellate‐ and Kupffer‐like cells. The authors treated these organoids with free fatty acids and found that the accumulation of lipids in the organoids and the degree of organoid steatosis were aggravated with increasing lipid content in the culture environment, and the organoids manifested inflammatory and fibrotic phenotypes. Gurevich et al.^194^ constructed steatosis organoid models with iPSC‐derived cryopreservable hepatocytes from patients with NASH. Intracellular lipid deposition in the organoids exhibited a dose‐dependent relationship with free fatty acids in culture, which viably conserved and recapitulated steatosis in in vitro conditions. Moreover, the organoids could be applied in drug metabolism research. Another study established bipotent ductal organoids from the hepatic explants of patients with NASH, which have impaired passaging/growth capability and NASH liver features.^178^ More recently, the group of Takebe cultured human liver organoids from 24 iPSC lines with various genotypes and assembled these organoids into a population organoid panel (PoP). After incubating with oleic acid, liver organoids of PoP showed the features of NASH. Through this model, the authors indicated that under different metabolic conditions, the glucokinase regulatory protein‐rs1260326 single nucleotide polymorphism could influence mitochondrial function, which is associated with NASH severity.^97^

Alcoholic liver disease (ALD) is one of the most common types of chronic liver diseases worldwide. The initial stage of ALD is alcoholic fatty liver, characterized by steatosis of liver cells. Some patients will develop into alcoholic steatohepatitis, liver fibrosis, and liver cirrhosis, which might eventually progress to liver cancer and liver failure.^195^ The ALD organoid model, reported by Wang et al.,^196^ was generated by treating human fetal liver mesenchymal cells/human ESC‐derived expandable hepatic organoids coculture system with ethanol. This model could simulate typical characteristics of ALD pathophysiology, including increased secretion of alanine aminotransferase, aspartate aminotransferase, and lactate dehydrogenase; decreased cell viability and apoptosis; increased CYP2E and CYP3A4 activity; enhanced oxidative stress; increased release of inflammatory cytokines; fibrosis; and increased deposition of ECM.

#### 4.1.4 Cancers

Currently, the most commonly used oncology models mainly include human tumor cell lines and patient‐derived xenograft (PDX) models. However, these models have some unavoidable shortcomings. Tumor cell lines involve primary (originated from patients) and immortalized tumor cells. Although primary cancer cells preserve some features of the parental tumor, the slow growth speed, short lifespan, and lack of complexity of tumors limit their applications. The immortalized cells have unlimited proliferation capacity, but they usually fail to represent the phenotypes and lose genetic heterogeneity of the original tumor during long‐term culture and passages, which may increase the failure rate of clinical drug screening trials.^197^ The 2D culture systems are unable to mimic the in vivo cellular growth conditions, and the 2D cultures fail to represent the tumor heterogeneity accurately. PDX models are constructed by transplanting human tumor cells into mice and promoting these cells to grow and form tumors. Xenograft models can maintain the relatively intact biological features of parental tumors as well as the 3D structure and tumor stroma. Studies have successfully generated several tumor PDX models, such as lung, colorectal, pancreatic, breast, prostate, and ovarian tumors.^198^, ^199^, ^200^, ^201^, ^202^ However, several shortcomings restricted them to being excellent preclinical models. Xenograft models are usually established from a small number of tumor tissues, which cannot completely inherit the genetic mutations from the primary tumor, and the stroma of the human tumor is gradually substituted by murine stroma with the PDXs growth.^203^ Many research results based on the PDX models have not been confirmed in human trials.^204^ Furthermore, the economic and time costs of PDX models are high with a low success rate.^24^ Tumoroids retain parental tumor heterogeneity and histopathological features and reserve their 3D structure after long‐term culture^32^, ^33^, ^205^, ^206^, ^207^ (Table 1). Papaccio et al.^208^ reported that patient‐derived colorectal tumoroids represented the morphology and immunohistochemistry characteristics and genomic and transcriptomic profile of corresponding tissues, and the response to anticancer drugs varied among these tumoroids derived from different patients.

**TABLE 1:** Comparison of the current preclinical models for cancer research.

	2D tumor cell lines	Patient‐derived xenografts	Tumoroids
Success rate of establishment	Generally high (but some special tumor types are low)	Relatively low	Relatively high
Maintenance	The easiest	Little difficult	Easy
Genetic manipulation	Able	Unable	Able
Genome‐wide screening	Able	Unable	Able
Relative cost	Low	High	Relatively high
High‐throughput assay	Able	Unable	Able
Expansion	Quick	Relatively low	Quick
Reproducibility	High	Moderate	Relatively low
Ability to recapitulate tumor development biology	Low	Low	High
Tumor immune microenvironment	Unable to recapitulate	Partial recapitulation	Partial recapitulation
Tumor heterogeneity	Unable to recapitulate	Retain, (but heterogeneity may be lost partly in long‐term culture)	Retain parental tumor heterogeneity
Ability of personalized treatment	Low	Moderate	High
Complexity	Low complexity	High complexity (enable to reproduce the cell types and the tumor cell–stroma cell interactions of the primary tumor and can be used to study metastasis)	High complexity (recapitulate histological and genetical features of primary tumors)

The role of mutations in genes or structures in tumor development is difficult to observe dynamically or to intervene. Therefore, the exploration of the tumorigenesis mechanism is usually performed with difficulties. Organoid technology enables in vitro simulation of the entire process of tumor development.^48^ According to organoid models, the accumulation of specific tumor‐driving gene mutations has been found to play a crucial role in tumorigenesis.^209^ The combination of genome editing and organoids offers new opportunities to study the role of pathogenic gene mutations. With CRISPR‐Cas9 technology, the oncogene c‐MYC associated with HCC was introduced into normal liver organoids.^210^ Excessive mitochondrial endoplasmic reticulum coupling occurred in these organoids and altered mitochondrial fission and aerobic respiration, finally resulting in HCC tumorigenesis. Human colorectal tumoroids have been utilized to discover three tumor suppressor genes (Acvr1b, Acvr2a, and Arid2) and identified the function of Trp53 in liver metastasis of colorectal cancer (CRC).^211^ Tumoroids serve as an advanced platform that is highly similar to the physiology and microenvironment features of in vivo tumors to propel our fundamental knowledge of tumorigenesis, progression, metastasis, and recurrence. The applications of organoids in modeling diseases are summarized in Table 2.

**TABLE 2:** Applications of organoids in disease modeling.

Organs or tissues	Diseases		Cell sources	References
Brain	Genetic diseases	Primary microcephaly	Human iPSCs and ESCs	148, 149
		Rett syndrome	Human ESCs and iPSCs	150
		Tuberous sclerosis complex	Human iPSCs	151, 152
		Fragile X syndrome	Human iPSCs	153
		Down syndrome	Human iPSCs	154
	Degenerative diseases	Parkinson's disease	Human iPSCs	45
		Alzheimer's disease	Human iPSCs	46
	Infection	ZIKV infection	Human ESCs and iPSCs	156, 220
		HSV‐1 infection	Human ESCs	156
		Human cytomegalovirus infection	Human iPSCs	163
		SARS‐CoV‐2 infection	Human iPSCs	174
	Tumor	Glioblastoma	Patient‐derived tumor cells	64, 121
		Medulloblastoma	Human iPSCs	65
		Glioma	Patient‐derived tumor cells	66
Retina	Genetic diseases	Nonsyndromic CLN3 disease	Human iPSCs	142
		Retinitis pigmentosa	Human iPSCs	143
		Leber congenital amaurosis	Human iPSCs	144, 145
	Tumor	Retinoblastoma	Human iPSCs and ESCs	236
Air ways	Genetic diseases	CF‐related air way diseases	Human ASCs	132
	Infection	Respiratory syncytial virus infection	Human ASCs	132
		SARS‐CoV‐2 infection	Human iPSCs and ASCs	10, 161, 174, 175
		Influenza virus infection	Human ASCs	161
		Cryptosporidium infection	Human ASCs	180
	Tumor	Lung adenocarcinoma	Patient‐derived tumor cells	71, 72
		Nonsmall‐cell lung cancer	Patient‐derived tumor cells	132, 216, 217
Gastrointestinal tract	Genetic diseases	CF‐related intestinal diseases	Human ASCs	134, 137
	Infection	SARS‐CoV‐2 infection	Human ASCs	10, 177
		*H. pylori* infection	Human iPSCs and ASCs	50, 182
		Human norovirus infection	Human ASCs	159, 160
		Cryptosporidium infection	Human ASCs	180
	Tumor	Neuroendocrine carcinoma	Patient‐derived tumor cells	33, 69
		Colorectal cancer	Patient‐derived tumor cells	38, 208, 216, 217, 232
		Gastric cancer	Patient‐derived tumor cells	184, 251
Hepatobiliary	Genetic diseases	Primary sclerosing cholangitis	Human ASCs	59
		α1‐Antitrypsin deficiency	Human ASCs	112, 113
		Alagille syndrome	Human ASCs and iPSCs	113, 114
		Wilson's disease	Dog ASCs	115, 116
		Wolman disease	Human iPSCs	117
		CF‐related bile duct disease	Human ESCs	135
	Metabolic diseases	Nonalcoholic fatty liver disease	Human iPSCs, ASCs	97, 127, 194
		Alcoholic liver disease	Human ESCs	196
	Infection	Rotavirus infection	Human ESCs and ASCs	162
		Viral hepatitis	Human iPSCs, ASCs, and ESCs	167, 168, 169, 170, 171, 172
		Salmonella Typhi infection	Mouse ASCs	186
	Fibrosis	Liver fibrosis	Human iPSCs	48
	Tumor	Cholangiocarcinoma	Patient‐derived tumor cells	15, 30, 33, 205, 206
		Gallbladder adenoma	Human ASCs	32
		Gallbladder carcinoma	Patient‐derived tumor cells	32, 33
		Bile duct ampullary adenocarcinoma	Patient‐derived tumor cells	38
		Hepatocellular carcinoma	Patient‐derived tumor cells and human iPSCs	205, 207, 233
Pancreas	Genetic diseases	Pancreatic dysplasia	Human iPSCs	48
		Wolfram syndrome	Human iPSCs	128
		Congenital hyperinsulinism	Human ESCs	129
	Tumor	Pancreatic ductal adenocarcinoma	Patient‐derived tumor cells	231, 258, 264
Thyroid	Autoimmune diseases	Hashimoto's thyroiditis	Human ASCs	58
		Graves’ hyperthyroidism	Human and mouse ASCs	60
	Tumor	Papillary thyroid cancer	Patient‐derived tumor cells	241
Kidney	Genetic diseases	Polycystic kidney disease	Human iPSCs	19
	Injury	Kidney injury	Human iPSCs	20
	Infection	SARS‐CoV‐2 infection	Human iPSCs	176, 179
	Tumor	Renal cell carcinoma	Patient‐derived tumor cells	257
Heart	Genetic diseases	Hypoplastic left heart syndrome	Human iPSCs	140
		Pregestational diabetes‐induced congenital heart defects	Human iPSCs	141
Salivary gland	Tumor	Salivary gland tumor	Patient‐derived tumor cells	38, 68
Mammary gland	Tumor	Breast cancer	Patient‐derived tumor cells	62, 198, 261
Prostate gland	Tumor	Prostate cancer	Patient‐derived tumor cells	214
Lacrimal gland		Dry eye disease	Mouse and human ASCs	226
Vasculature	Metabolic diseases	Diabetic vasculopathy	Human iPSCs	47
Blood–brain barrier	Infection	*Plasmodium falciparum* infection	Human ASCs	181
		Lyme neuroborreliosis	Human ASCs	182

ASCs, adult stem cells; CF, cystic fibrosis; ESCs, embryonic stem cells; iPSC, induced pluripotent stem cells; SARS‐CoV‐2, severe acute respiratory syndrome coronavirus 2; ZIKV, Zika virus.

**FIGURE 4:**
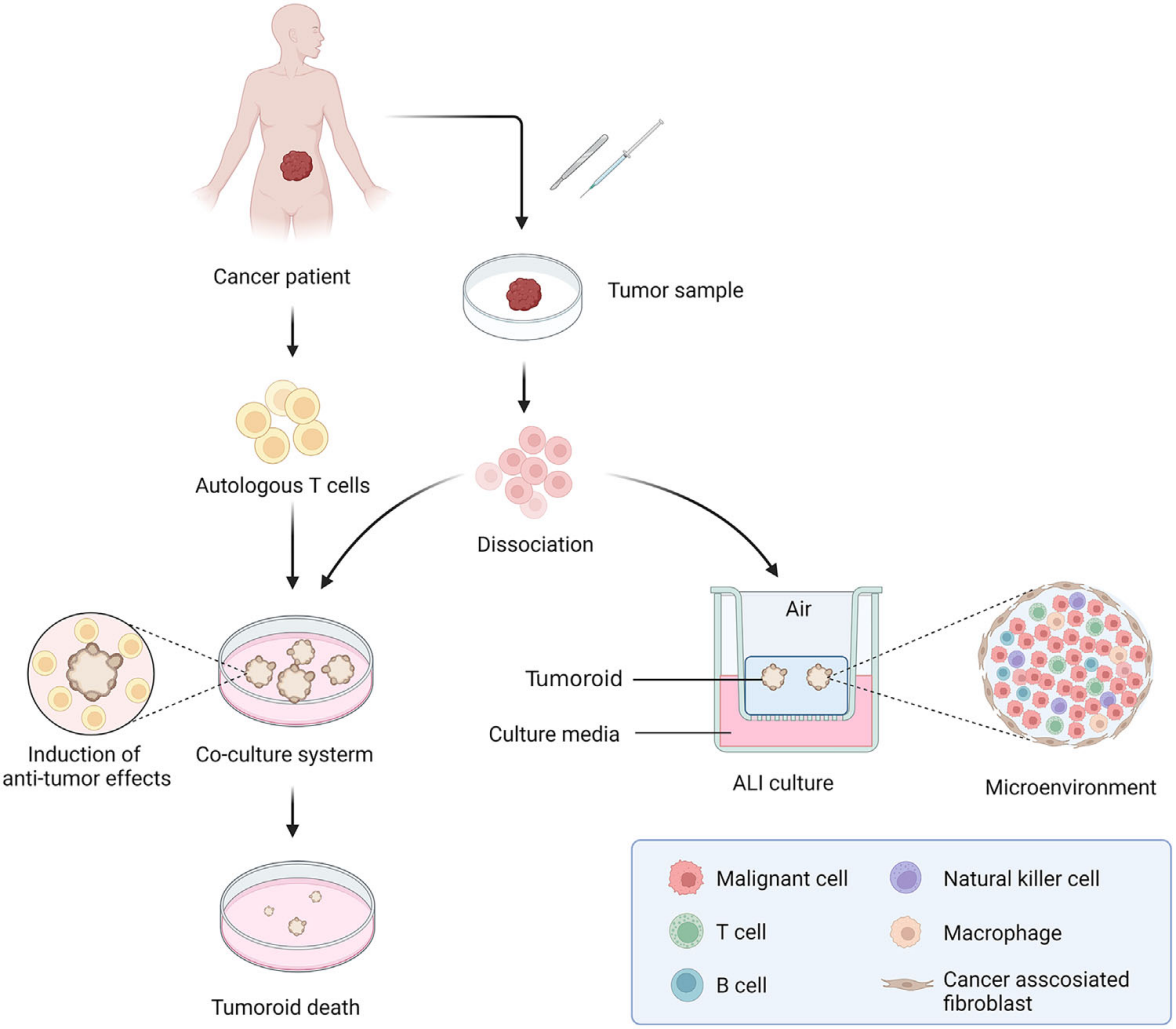
Recapitulation of tumor immune microenvironment with tumoroids. Tumor samples are obtained by resection or biopsy and dissociated into single cells to form tumoroids. Tumoroids are established from tumor cells and cocultured with autologous T cells by submerged method, which can induce the specific antitumor effects of T cells, leading to the injury and death of tumoroids. With the application of ALI method, the tumor tissues are used as the initial cell materials to generate tumoroids preserving cancer‐associated fibroblasts and immune cells, which can recapitulate the complex tumor microenvironment in vitro (Created with BioRender.com).

It has been widely accepted that the tumor immune microenvironment plays an essential role in tumor formation and progression. Based on the cancer immunoediting theory, the immune system can inhibit or promote tumor growth in different stages.^212^ Despite advances in immunotherapy, a part of patients present with low responses to this treatment. One of the hot topics in the field of tumor immunotherapy is the identification of patients who are sensitive to immunotherapy. Evidence has shown that the tumor immune microenvironment is recapitulated via coculturing tumoroids and stromal cells such as TILs and cancer‐associated fibroblasts (CAFs)^213^ (Figure 4). Zhang et al.^214^ incubated tumoroids derived from prostate cancer patients and CAFs and found that neuregulin 1 released by CAFs could activate human EGF receptor 3 in cancer cells, leading to antiandrogen therapy resistance. Kuo et al. successfully generated patient‐derived tumoroids from 100 patients with 28 distinct tumor subtypes with ALI culture system, which retained a variety of immune cells such as T cells, B cells, NK cells, and macrophages, and the T cell receptor spectrum of parental tumors. However, with time going on, the immune components of ALI‐cultured tumoroids would decrease. Treatment with nivolumab in these tumoroids could screen for specific patients who respond to immunotherapy.^38^, ^215^ The interactions between tumor cells and T cells have been modeled by tumoroid and peripheral blood mononuclear cell (PBMC) coculture systems. Tumor‐reactive T cells were generated by coculturing PBMCs and tumoroids, and they have tumor‐specific T cell responses.^216^, ^217^ Liu et al.^218^ established autologous organoid‐ killing models to confirm that high‐affinity neoantigens had higher antitumor activity in HCC patients. With a similar method, a recent RCT study (NCT03026140) demonstrated that there existed an association between induced T cell reactions in vitro and patient response.^219^

### 4.2 Drug research

Organoids have distinct advantages in that they can recapitulate in vivo physiological functions and features of organs or tissues, excelling in a wide range of models for drug‐related research. As described above, ZIKV infection contributes to birth defects and miscarriage. A work by Li et al.^220^ demonstrated that methylene blue protected brain organoid cells from ZIKV infection via blocking viral protease NS3 and NS2B interactions. Another recent study represented the HEV–host interactions in liver organoids and reported that brequinar and homoharringtonine are potent anti‐HEV drugs.^171^ Bacterium‐infected intestinal organoids have been utilized to serve as advanced platforms for drug discovery. Bacitracin, an antibiotic for local application, has been identified to inhibit the activity of Clostridium difficile and its toxin B.^221^ Islet organoids were used for drug screening in diabetes.^222^ Based on 3D culture, GLIS3−/− human ESCs (hESCs) were induced to act as a high‐throughput platform for drug identification in GLIS3‐associated diabetes.^223^ High glucose metabolism could stabilize and activate HIF‐1α, and upregulate some HIF‐1α target genes. PX‐478, a HIF‐1α inhibitor, was employed to treat human islet organoids with chronic high glucose exposure, enabling the glucose‐induced insulin secretion stimulation index increase, which was considered as a potential antidiabetic agent.^224^ Moreover, the lacrimal gland organoids are reported as promising tools that allow high‐content drug screening.^225^, ^226^

Patient‐derived organoids are advanced models for discovering and testing novel drugs.^227^ Based on the results of drug sensitivity tests, patients are categorized to find the similarities in genetic mutations or epigenetics in those who respond to specific therapeutic regimens, which may help advance the precision of antitumor treatment.^228^, ^229^ The L1 cell adhesion molecule (L1CAM) is a trigger factor of metastasis and is upregulated in tumor that have undergone neoadjuvant chemotherapy. Ganesh et al.^230^ indicated that knockdown L1CM human colorectal tumoroids presented improved sensitivity to irinotecan. The coculture system of pancreatic ductal adenocarcinoma (PDAC) organoids and CAFs showed resistance to gemcitabine, 5‐FU, and paclitaxel, with increased epithelial‐to‐mesenchymal transition (EMT) gene expression in these organoid cells.^231^ Colorectal tumoroids were used to perform drug sensitivity assays and pharmacogenomics analysis, and results showed that the paclitaxel sensitivity was linked to the checkpoint with forkhead and ring finger domains (CHFR) silencing in organoid as well as in xenograft models.^232^ Organoids can be cultured in a short timescale with high generation efficiency, which is valuable for extending the applicable scope of existing medicines. CF patient‐derived organoids were employed in a high‐throughput compound assay to identify potential drugs to treat individuals having rare CFTR mutations.^137^ Yuan et al.^32^ performed a drug sensitivity test for 29 antitumor compounds approved by the United States Food and Drug Administration (US FDA) in human gallbladder tumoroids, and histone deacetylase (HDAC) inhibitors, including vorinostat and curcumin, significantly suppressed the growth of GBC tumoroids. Furthermore, CUDC‐907, a dual PI3K/HDAC inhibitor, presented a higher level of tumor suppressive activity. PCSK9 has been reported as a therapeutic target for patients with dyslipidemia or hypercholesterolemia, and overexpression of PCSK9 is associated with oncogenesis and enhances the malignant phenotypes of CRC with APC/KRAS mutations. Wong et al.^233^ found that blocking PSCK9 significantly suppressed the growth of human APC/KRAS colorectal tumoroids. The protein synthesis inhibitor omacetaxine has been approved by the US FDA to treat patients with chronic myelogenous leukemia.^234^ However, in a recent study, this drug was proven to be a highly effective compound that could repress the growth of HCC patient‐derived organoids and promote tumoroid cell apoptosis. Thus, omacetaxine has the potential to be a new option for HCC treatment.^235^ Watanabe et al.^236^ established human PDAC tumoroids for drug screening, and the CHK inhibitors exhibited effective antitumor ability, which was found to be effective against breast and ovarian cancers and has been proven in clinical trials (NCT03495323, NCT02203513). Sunitinib has been approved to treat kidney cancer and imatinib‐resistant gastrointestinal stromal tumors. At present, sunitinib has been identified as a potential agent for the treatment of retinoblastoma with minimal toxicity to normal retinal cells.^237^

Advances in organoid technologies allow for the development of efficient therapies or combination regimens. The nicotinamide adenine dinucleotide phosphate oxidase (NOX) can produce ROS to promote EMT of cells, eventually causing cancer, and NOX4 plays an important role in the development of various tumors.^238^ Fangchinoline, a small molecule extracted from Menispermaceae, was found to exert antitumor effects in nonsmall cell lung cancer (NSCLC) organoids via inhibition of NOX4 to reduce ROS, hamper Akt‐mTOR pathway activation and decrease EMT and malignant phenotypes.^239^ Ghate et al treated patient‐derived colon tumoroids with the VprBP inhibitor B32B3 and found that B32B3 could restrain H2AT120 phosphorylation and restart the normal transcriptional program to impair the proliferation activity of colon tumoroids.^240^ BRAFV600E inhibitor monotherapy was employed to treat human papillary thyroid tumoroids with the BRAFV600E mutation and exhibited mild antitumor activity. On the basis of BRAF inhibitors, combining MEK, RTK inhibitors, or chemotherapies could remarkably decrease the viability of tumoroid cells.^241^ AT‐rich interaction domain 1A (ARID1A) is one of the subunits of the BRG1‐ or HBRM‐associated factor complex and has been demonstrated to have tumor suppressor effects.^242^, ^243^ ARID1A mutation or deficiency has been shown to contribute to facilitating the aggressiveness of tumors,^244^ and it has been identified that most gastric cancer patients carry ARID1A mutations. Recent research by Loe et al.^245^ provided a novel combination therapy of TP06 (epigenetic inhibitor) and Nutlin‐3 (p53 agonist) to treat gastric tumoroids with Arid1a heterozygosity, exhibiting a robust inhibition of tumor growth.

Other applications of organoids in drug‐related research also include toxicity assessment and drug safety evaluation. The liver and kidney are the primary organs and play essential roles in the processes of drug metabolism and excretion. Therefore, they are vulnerable to drug‐related injuries. The traditional models for toxicity assessment include 2D cell lines and animal models; however, these models have always suffered from some insurmountable drawbacks.^246^, ^247^ In addition, the expression and function of enzymes and proteins related to drug metabolism as well as metabolic functions, vary between species, making it impossible to accurately assess drug toxicity.^246^ Organoids highly resemble the structure and metabolism characteristics of original organs or tissues, which provide excellent preclinical models for toxicity assessment and drug safety evaluation. Under the condition of differentiation into the hepatic lineage, human ASC‐derived intrahepatic cholangiocyte organoids (ICOs) can represent the metabolism features of the liver, such as the expression of CYP enzymes.^123^ Shi et al.^248^ identified the associations between necroptosis and bile duct disease via ICOs, which could serve as a useful ex vivo model for biliary cytotoxicity assessment. Bouwmeester et al.^249^ established hepatocyte‐like ICOs and detected the expression and activity of drug metabolism‐related genes to confirm the ability of organoids in ex vivo toxicity assessment. Zhang et al.^250^ developed a high throughput screening platform based on the human liver organoid‐on‐a‐chip for drug‐induced liver injury that pooled multiomics such as biomarker/analyte detection, high‐content imaging‐enabled phenotyping, and single‐cell RNA sequencing. The authors demonstrate the effectiveness of this platform by testing a set of known hepatotoxic drugs and comparing the results to clinical data. Another study demonstrated the potential utility of hepatic organoids for hepatotoxicity and drug safety assessment by conducting a functional analysis of CYP450‐mediated metabolic ability.^251^ To test the potential nephrotoxicity of Esculentoside A, Gu et al.^252^ exposed iPSC‐derived kidney organoids to the compound and monitored changes in cell viability, morphology, and gene expression. They found that exposure to Esculentoside A resulted in decreased cell viability and altered gene expression patterns, indicating that organoids offered a promising approach for assessing the toxic effects of compounds. What's more, the side effects of most anticancer compounds involve gastrointestinal symptoms such as abdominal pain, vomiting, nausea, and diarrhea. Serious gastrointestinal reactions may force the patient to discontinue treatment, leading to a dismal survival outcome and poor quality of life. However, the underlying mechanisms of gastrointestinal toxicity caused by many antineoplastic drugs have not been fully clarified. Recent studies forecasted the gastrointestinal toxicity caused by gefitinib and doxorubicin by evaluating the viability and apoptosis of the colon and small intestine after exposure to the two compounds. Furthermore, the possible molecular mechanisms of gefitinib and doxorubicin‐triggered intestinal toxicity were explored by transcriptomic analysis.^253^, ^254^

In conclusion, organoids are an important tool for assessing the effects and toxicity of drugs, understanding drug metabolism and distribution, predicting drug safety and efficacy, and providing better guidance for drug development and clinical application. The use of organoids in drug research has revolutionized the drug development process. Organoids provide a powerful tool for modeling human physiology and disease, which can improve drug discovery, reduce the failure rate of clinical trials, and accelerate the development of new therapies.

### 4.3 Precision medicine

Organoids have shown great potential in the field of precision medicine, which aims to tailor medical treatments to individual patients based on their specific genomics and metabolomics.^255^ By providing a more physiologically relevant and personalized model of human organs or tissues, organoids can be used to predict individual patient responses to drugs and other treatments.^256^, ^257^

One potential application of organoids in precision medicine is in the development of personalized cancer treatments. PDOs can be used to test the efficacy of different chemotherapy drugs and identify the most effective treatment for that patient.^258^, ^259^ This approach is more accurate than traditional methods of testing drugs on cancer cell lines, which do not fully capture the genetic diversity of individual tumors. An article by Khan et al. described the application of organoids to predict the response of patients presenting with metastatic digestive system tumors to various chemotherapy drugs and targeted drugs. The researchers found that the organoids accurately forecasted which treatments would be effective in the patient and which would not, with significantly high specificity (93%) and sensitivity (100%).^260^ Similar work was carried out by Yao et al., who established locally advanced rectal cancer patient‐derived organoids to construct a biobank. The study demonstrated that rectal tumoroids could recapitulate the patient's response to neoadjuvant chemoradiation in vitro, with an accuracy rate of 86%, and also was able to identify patients who were likely to have a complete response to chemoradiation therapy, allowing clinicians to tailor treatment to the individual patient.^261^ According to the study by Lyudmyla, the drug sensitivity results of PDAC patient‐derived organoids were highly consistent with the clinical response of patients, and the PDO forecasted drug response was also linked to tumor cellularity.^262^ Furthermore, studies have identified the feasibility that PDOs could serve as platforms for fast drug screening and allowed patients to achieve the most appropriate therapeutic regimens.^262^, ^263^ It is worth noting that, in addition to directly guiding patients on medication, the overall analysis of tumoroids can be applied in the discovery of biomarkers for clinical treatment response. The team of Burkhart suggested that the clinical sequencing results of PDAC patients who have better responses to chemotherapies tended to have fewer mutations in KRAS, TP53, and SMAD4 and the specific pharmacotype of PDOs had potential as a forecasted biomarker for the response of PDAC patients to chemotherapies via an ongoing clinical trial (NCT03563248).^264^ Target therapy is a precision medicine approach that targets specific molecular markers or signaling pathways of tumors and selects specific drugs for treatment.^265^ Targeted therapy is a part of precision medicine that uses specific compounds to inhibit specific molecular markers or signaling pathways of tumors or other diseases. Organoids are also used to perform target drug screening and discover potential therapeutic targets owing to the important roles of organoids in target therapy theories.^100^, ^266^ Ovarian tumor organoids derived from patients with three tumor types had been used as a high‐throughput screening platform to test the sensitivity to 240 protein kinase inhibitors by Phan et al.,^267^ and this platform allowed rapidly obtaining drug sensitivity results for ovarian cancers. Yuan et al.^32^ screened 20 US FDA‐approved targeted drugs with low toxicity to normal gallbladder organoids and found that HDAC inhibitors could significantly decrease the growth of the gallbladder tumoroids. After further study, they demonstrated that the dual phosphatidylinositol‐4,5‐bisphosphate 3‐kinase (PI3K) and HDAC inhibitor CUDC‐907 had higher antitumor activity than the HDAC inhibitor alone in the gallbladder tumoroid and xenograft mouse models.^32^ A novel organoid culture system combining suspension and rotating culture methods was developed to cultivate colorectal tumoroids on a large scale for drug screening with high accuracy, robustness, and reproducibility because this method could improve the scalability and decrease the variability of organoids. The study tested 56 anticancer agents, of which 47 were inhibitors with small molecules that targeted specific tumor‐associated pathways, and suggested that the tumor drug response heterogeneity was attributed to the diversity of genes or epigenetics. Furthermore, based on the gene−drug analysis, a significant correlation between the BRAFV600E mutation and vemurafenib was observed as well as TP53 mutations and the MDM2 inhibitor nutlin‐3.^232^ Organoids present with the potential as promising tools or models to explore the underlying drug resistance mechanisms and help customize the optimal treatment plan. Zhao et al.^268^ analyzed hepatobiliary tumoroids with various drug susceptibilities using single‐cell transcriptome sequencing technology. CD44+ hepatoma cells were found to have the ability of broad‐spectrum drug resistance and metabolic advantages. Lee et al. found that SHP099, an inhibitor of Src homology 2 domain‐containing phosphatase 2 (SHP2), could abrogate receptor tyrosine kinase (RTK)‐induced reactivation of the MAPK/ERK kinase (MEK)/extracellular regulated kinase (ERK), and protein kinase B (AKT) signaling pathways to eliminate sorafenib resistance.^269^ To verify this result, they treated HCC patient‐derived organoids with SHP099 and sorafenib and showed that the combination treatment significantly inhibited tumor cell growth and that the HCC cells in these organoids were sensitive to sorafenib again. ML264, a Kruppel‐like factor 5 (KLF5) inhibitor, was found to be a suppressor of the KLF5/Bcl‐2/caspase3 signaling pathway and can restore the sensitivity to oxaliplatin in oxaliplatin‐resistant colorectal tumoroids by the inhibition of antiapoptotic effects.^270^

Organoids can also be used to study rare genetic diseases and identify personalized treatments. By deriving organoids from patients with genetic diseases, researchers can study the effects of different drugs and gene therapies on these tissues, and develop tailored treatments for individual patients. Sampaziotis et al.^271^ applied 3D culture technology to construct CF organoid models from iPSC‐derived cholangiocytes and explored the response of the CF organoids to VX‐809 CF therapy. As mentioned above, FIS is considered as a prospective biomarker to predict the response to CFTR‐modulating drugs. Technological advances in genetic engineering, especially CRISPR–Cas9 genome editing, allow gene repair to be a promising treatment for some diseases, and organoids provide a good platform to develop and validate novel gene therapies.^272^ Schwank et al.^273^ pioneered the successful application of CRISPR/Cas9 to human‐derived organoids. They restored the CFTR function of intestinal organoids by correcting the common mutation F508del in CF. More recently, Geurts et al.^274^ employed CRISPR‐based adenine base editors that enabled A‐T to G‐C base changes to successfully correct the W1282X and R553X mutations and achieve functional CFTR rescue in human rectal and airway organoids. A clinical trial (NCT04254705) evaluates the response to CFTR‐modulators in intestinal organoids of CF patients with R334W mutations.

Finally, organoids can be used to study the effects of environmental toxins and other factors on human tissues. By exposing organoids to different environmental conditions, researchers can study the effects of air pollution, toxic chemicals, and other factors on human health, and identify personalized strategies for reducing exposure and preventing disease. For example, Kim et al.^275^ exposed human iPSC‐derived alveolar organoids to diesel PM2.5 to investigate its developmental toxicity, and the authors showed that diesel PM2.5 could upregulate the oxidative stress and EMT expression to impair alveolar epithelium growth and contribute to high susceptibility to SARS‐CoV‐2. The lung toxicity of common air pollutants including benzo(a)pyrene, nano‐carbon black, and nano‐SiO2 has been assessed with human iPSC‐derived AT2‐like cell organoids.^276^ Meanwhile, human airway organoids were employed as models for toxicological assessment of emerging inhaled pollutants of tire wear particles.^277^ Nicotine exposure in pregnant women has been shown to cause harmful birth outcomes and effects on cerebral development.^278^ Several studies have investigated the role of nicotine in abnormal human brain development and the potential mechanisms with organoid system models.^279^, ^280^ Heavy metals are pervasive and persistent environmental toxins with the feature of accumulation in organisms, which may lead to multiorgan damage. The liver and cardiac organoids have been used to evaluate the toxic effects of lead, mercury, and thallium, and these heavy metals were found to inhibit the heart rates of heart organoids.^281^ Through the mouse intestinal organoid model, it was found that excessive intake of cadmium could activate the Notch pathway, increase the synthesis of ROS, cause intestinal mucosal damage, and more susceptible to Salmonella infection.^282^ In addition, the brain organoids treated with Cd exhibited increased neuron apoptosis, disrupted proliferation of neural progenitor cells, and change gene expression profiles, leading to neuroinflammation and ciliogenesis restriction.^283^ However, Cd was not prone to hamper the neural differentiation of cerebral organoids. Organic pollutants with chemical toxicity are difficult to be degraded, and tend to accumulate in organisms in various ways, causing dysfunction and failure of human organs.^284^ Evidence has pointed out that polybrominated diphenyl ethers are more likely to disrupt the differentiated NPCs in an hESC neural differentiation model and exert their cytotoxicity relying on the developmental stage.^285^ Bisphenols are widely used to produce epoxy resins, and polycarbonate plastics, which have been shown to have endocrine disruptor effects and developmental toxicity in mammalian embryos.^286^, ^287^ Recently, the retinotoxicity of BPA, TBBPA, and TBBPS has been evaluated with human retinal organoids derived from ESCs.^288^ It was reported that all three bisphenols could adversely affect retinal organoid development and that TBBPS had higher toxicity. Furthermore, bisphenols induced retinotoxicity in a time‐dependent manner. Meanwhile, exposure to BPS and BPF, the alternative chemicals of BPA, could increase the branches and alter the protein expression of mammary organoids, which differed from those induced by estrogen and might raise the risk of breast cancer occurrence.^289^ Nanomaterials can be utilized as powerful and flexible tools for the diagnosis and treatment of human diseases as well as engineering and environmental protection fields. However, nanoparticles have been pointed to lay an adverse influence on the nervous, respiratory, circulatory, and reproductive systems and could increase the risk of malformations and cancer.^290^, ^291^, ^292^, ^293^, ^294^, ^295^ Organoid models open a new door for studying the toxicological effects of nanomaterials. Mekky et al.^296^ offered confirmation of the feasibility of liver organoids for the hepatotoxic assessment of Mg nano. The work by Zuo's team developed kidney organoids as a toxicity screening platform to test black phosphorus quantum dots.^297^ Overall, the use of organoids in precision medicine has the potential to revolutionize the way we develop and deliver medical treatments, by providing more accurate and personalized models of human tissues that can be used to predict individual patient responses to different drugs and environmental factors. Given the enormous potential of organoids in biomedical applications such as drug discovery, efficacy and safety testing, and clinical personalized medicine, several organoid‐based preclinical or clinical trials are already registered or underway (Table 3).

**TABLE 3:** Summary of organoid‐based clinical trials registered in the ClinicalTrials.gov database.

Application	Condition or disease	Trial number	Phase	Recruitment Status	Study type	Treatment
Drug screening /Personalized treatment	Cystic fibrosis	NCT04254705	N/A	Unknown	CT	Tezacaftor + Ivacaftor
	Advanced inoperable abdominal tumors	NCT05378048	2	Not yet recruiting	RCT	Antitumor therapy
	Refractory solid tumors	NCT04279509	N/A	Unknown	CT	Chemotherapy
	Cholangiocarcinoma	NCT05644743	N/A	Not yet recruiting	PSO	Gemcitabine + cisplatin
		NCT05634694	N/A	Recruiting	PSO	Chemotherapy
	Breast cancer	NCT03544047	N/A	Unknown	CT	Paclitaxel
		NCT03925233	N/A	Unknown	RSO	Antitumor drugs
	Colorectal cancer	NCT04996355	N/A	Recruiting	PSO	Chemotherapy
		NCT03577808	N/A	Unknown	PSO	Neoadjuvant chemoradiation
		NCT05352165	N/A	Not yet recruiting	RCT	Neoadjuvant therapy
		NCT05304741	N/A	Recruiting	PSO	Chemotherapy and targeted agents
		NCT05725200	N/A	Recruiting	CT	Antitumor drugs
		NCT04906733	N/A	Recruiting	PSO	Chemotherapy with/without cetuximab
	Lung cancer	NCT03979170	N/A	Recruiting	PSO	Chemotherapy
		NCT05669586	2	Recruiting	RCT	Antitumor therapy
	Ovarian cancer	NCT05175326	N/A	Recruiting	PSO	Chemo‐ and targeted therapy
		NCT05290961	N/A	Recruiting	PSO	Antitumor drugs
	Recurrent high grade astrocytic glioma	NCT05532397		Not yet recruiting	CT	Antitumor drugs
	Esophageal cancer	NCT03283527	N/A	Unknown	PSO	Chemoradiation
	Pancreatic cancer	NCT05196334	N/A	Recruiting	PSO	Antitumor therapy
		NCT03544255	N/A	Unknown	PSO	Antitumor therapy
		NCT04931381	N/A	Recruiting	RCT	Chemotherapy
	Gastric cancer	NCT05351398	N/A	Not yet recruiting	RCT	Neoadjuvant therapy
	Gastro‐intestinal cancer	NCT05652348	N/A	Recruiting	PSO	Hyperthermic intraperitoneal chemotherapy
	Nonmuscle‐invasive bladder cancer	NCT05024734	2	Recruiting	CT	Epirubicin, mitomycin, gemcitabine, docetaxel
Prediction of treatment response	Colorectal cancer liver metastasis	NCT05183425	N/A	Recruiting	–	Antitumor drugs
	Pancreatic cancer	NCT04736043	N/A	Recruiting	–	Antitumor drugs
		NCT04777604	N/A	Not yet recruiting	–	Neoadjuvant treatment
	Ovarian cancer	NCT04555473	N/A	Recruiting	–	Primary debulking surgery (PDS) + adjuvant chemotherapy and neoadjuvant chemotherapy + interval debulking surgery
	Gastric cancer	NCT05203549	N/A	Recruiting	–	Neoadjuvant therapy
	Breast cancer	NCT05007379	N/A	Not yet recruiting	–	New CAR‐macrophages
	Advanced refractory cancers	NCT05267912	N/A	Recruiting	–	Chemotherapy, hormonal therapy, targeted therapy
	Intestinal irradiation and inflammatory bowel disease	NCT05425901	N/A	Not yet recruiting	–	Irradiation
	Nonsmall cell lung cancer	NCT05136014	N/A	Enrolling by invitation	–	Tyrosine kinase inhibitors
		NCT04826913	N/A	Not yet recruiting	–	Chemotherapy (cisplatin, carboplatin, pemetrexed), targeted therapies or immunotherapy and radiotherapy
		NCT05332925	N/A	Recruiting	–	Immunotherapy
	Head and neck cancer	NCT05400239	N/A	Not yet recruiting	–	Chemoradiotherapy
Biobank	Triple‐negative breast cancer	NCT05404321	N/A	Recruiting	–	–
	Breast cancer	NCT05317221	N/A	Recruiting	–	–
	Neuroendocrine neoplasm	NCT04927611	N/A	Recruiting	–	–
	Colorectal cancer metastases and hepatocellular carcinomas	NCT05384184	N/A	Recruiting	–	–
	Liver, biliary, and pancreatic cancer	NCT02436564	N/A	Unknown	–	–
	Kidney cancer	NCT04342286	N/A	Completed	–	–
	Intrauterine adhesion	NCT05521932	N/A	Not yet recruiting	–	–
	Head and neck cancer	NCT04261192	N/A	Recruiting	–	–
	Digestive system diseases (inflammatory bowel disease, ulcerative colitis type)	NCT05294107	N/A	Recruiting	–	–
	Hematologic malignancy	NCT03890614	N/A	Recruiting	–	–
	Glioma tumor	NCT04865315	N/A	Active, not recruiting	–	–
	Lung cancer	NCT04859166	N/A	Completed	–	–
	Cancers	NCT05734963	N/A	Recruiting	–	–
	Vaginal HPV infection	NCT04278326	N/A	Recruiting	–	–
	Pancreatic cancer	NCT05727020	N/A	Recruiting	–	–
Models for basic research	Host–microbe interaction	NCT05323357	N/A	Recruiting	–	–
	Simultaneous establishment of PDAC organoids and CAFs	NCT05571956	N/A	Recruiting	–	–
	The role of the immunological microenvironment in chemoresistant colorectal cancer	NCT05038358	N/A	Not yet recruiting	–	–
	Characterize the level of proliferation of high‐grade astrocytoma organoids	NCT03971812	N/A	Unknown	–	–
	The biology of innervated sensory epithelial cells	NCT02888587	N/A	Completed	–	–
	The mechanisms of aggressive tumor growth and treatment resistance in glioblastoma	NCT04868396	N/A	Active, not recruiting	–	–
	Grafts of patient‐derived glioblastoma stem cells onto autologous brain organoids for testing drugs against tumor invasion	NCT05772741	N/A	Recruiting	–	–
	Human‐specific mechanisms of preimplantation embryo development and early pregnancy	NCT05231551	N/A	Recruiting	–	–
	Characterization of meningioma patient‐derived organoids	NCT04478877	N/A	Recruiting	–	–
	Characterization of primary sclerosing cholangitis patient‐derived organoid	NCT04753996	N/A	Recruiting	–	–
	Molecular characterization of metastatic prostate cancer	NCT05577689	N/A	Not yet recruiting	–	–
	Comparison of basic properties of gut epithelia of hypertensive and normotensive reference subjects	NCT04497727	N/A	Active, not recruiting	–	–
	Characterization of intestinal stem cells	NCT02874365	N/A	Recruiting	–	–
	Mechanisms of food sensitivity	NCT03256266	N/A	Recruiting	–	–
		NCT05259826	N/A	Recruiting	–	–

CAFs, cancer associated fibroblasts; CT, clinical trial; N/A, not available; PDAC, pancreatic ductal adenocarcinoma; PRO, prospective observational; RSO, retrospective observational.

*Data source*: ClinicalTrials.gov website (https://clinicaltrials.gov/ct2/home).

### 4.4 Regenerative medicine

Regenerative medicine refers to biological and engineering methods to repair, regenerate, or replace damaged or absent cells, tissues, or organs to restore normal structure and function. Organ transplantation is crucial for treating patients suffering from organ failure, but it faces various challenges, such as severe graft rejection and a growing shortage of donors. To overcome these obstacles, in vitro‐cultured organoids have emerged as a promising alternative, offering an unlimited supply of potential donors for autologous transplantation therapy and tissue repair. Several experiments have clarified that organoids transplanted into animals could restore impaired organ function.^298^, ^299^, ^300^, ^301^ Yang et al.^302^ constructed bioprinted hepatorganoids using 3D printing technology and transplanted them into immunodeficient mice with tyrosinemia type I and liver failure. The organoids could form functional vasculature systems after transplantation. The serum levels of liver function biomarkers significantly declined, indicating alleviation of liver injury and an apparent therapeutic effect of hepatorganoids on tyrosine metabolism defects. Implantation of pancreatic islet‐like organoids generated by planting pancreatic islet cells into a bioscaffold has the ability to restore insulin secretion and decrease glucose levels in type 1 diabetic mice.^303^ Human brain organoids could keep survival, form long lateral hypothalamus projections and functionally incorporate into the brain circuits after transplantation into the medial prefrontal cortex of mice.^304^

Moreover, there are still some patients presenting with the dysfunction or defects of certain tissues or organs such as biliary atresia, bile tract injury, and short bowel syndrome due to congenital and acquired factors, for which effective therapeutic regimens are usually unavailable. The emergence and advancement of organoid technology provides powerful support for the generalization and application of tissue repair and regeneration. Sampaziotis et al.^305^, ^306^ pioneered the use of human bile duct epithelial organoids to repair the gallbladder and bile duct of mice. Cholangiocyte organoids were generated using normal human bile duct tissue. After being marked by a green fluorescent protein, these organoids were seeded on polyglycolic acid or densified collagen scaffolds to repair the gallbladder or bile duct of immunodeficient mice. The results showed that the mice transplanted with cholangiocyte organoids had extended survival, and their bile ducts were unobstructed, without manifestations of obstructive jaundice and increased bilirubin and alkaline phosphatase. More recently, a study injected human‐derived gallbladder organoids marked by red fluorescent protein into the intrahepatic ducts of isolated human livers.^307^ The authors discovered red fluorescent cells in the injected bile ducts, without duct dilatation or obstruction, and the transplanted cholangiocytes expressed key biliary markers. The results implied that organoids can be applied to repair human bile ducts. Similar findings were described in a study by Roos et al.^308^ Bile cholangiocyte organoids rapidly repopulate decellularized extrahepatic biliary duct scaffolds and restore the monolayer of cholangiocyte‐like cells in vitro. Jejunum organoids derived from 12 kids with intestinal failure were seeded into human intestinal scaffolds and able to differentiate into columnar epithelial cells with crypt units that fully covered the scaffolds, which could reappear several physiological jejunal functions.^309^ Mouse transplantation experiments exhibited that these grafts survived and built a luminal structure. Similarly, Sato's team recently reported a small intestinalized colon (SIC) generated by transplanting small intestinal epithelium onto the colon surface.^310^ The authors suggested that SIC could form the nascent villus and subepithelial lymphatic structures that are only observed in the small intestine. Furthermore, in SBS rats, xenotransplanted SIC could establish a complete vascular system and exert small intestine function, leading to improved survival and a low risk of intestinal failure.

## 5 LIMITATIONS AND FUTURE PERSPECTIVES

The past decade has witnessed dramatic progress in organoid technologies. Organoids have unique strengths in that they mimic nearly all physiological conditions and conserve parental genetic stability. They can be generated from a few cells or tissues for disease modeling and drug screening studies and can be used to reverse the disease‐causing mutation to treat disorders resulting from mutations. Furthermore, organoids have fast growth and a high culture success rate, which might solve the problem of the low tumor‐forming efficiency of patient‐derived tumor xenograft models. However, the technology is not yet mature, and many hurdles still exist that need to be overcome.

First, only a few laboratories are able to perform organoid culture, and experimental repeatability between different research groups is low. The reason is that the development of organoids requires the timely activation of morphogenetic signaling pathways to induce cell fate and promote the separation of different types of cells, thereby jointly completing the self‐organization process. Therefore, it is necessary to add a variety of growth factors or nutritional components to simulate the microenvironment of cell growth in vivo and use corresponding techniques to maintain the unique 3D spatial structure of the organoids. However, the high price of growth factors and medium additives restrict the popularization of organoid culture technology, and the types and doses of growth factors and nutrients used in organoid culture media also vary from laboratory to laboratory. The addition of a variety of growth factors might also cause gene mutations, resulting in a gap between the results of mechanism exploration or drug susceptibility testing and the real situation. The batch and quality of original cells and the changes in gene expression and transcriptomics in the culture and passage process also contributed to confounding effects.^311^ Krefft et al.^312^ proposed a protocol that enabled the generation of forebrain cerebral organoids with low heterogeneity by controlling the patterning factors of iPSCs. The matrix softening method was employed to specify the organoid geometry to control their patterning, achieving spatiotemporal control over organoids.^313^ Future efforts should be devoted to developing inexpensive and high‐quality media and establishing procedures and quality control standards to increase the popularity of organoid technologies and the reproducibility of organoid generation.

Second, another factor hindering the organoid from being an excellent tissue biology model is its limited cellular development and low cell maturity (especially for PSC‐derived organoids). PSC‐derived organoids resemble fetal tissue rather than adult tissue. Two reasons are involved in this phenomenon: on the one hand, organoids are unable to develop to the maturation stage because of their short life span. As the organoid volume increases, the demand for organoids to acquire nutrients and oxygen and expel metabolites cannot be met, which results in cell death and tissue necrosis because of hypoxic metabolite accumulation. Consequently, current organoids cannot replace actual tissues and organs in terms of size and physiological function. The application of bioreactors may solve this problem, which can improve nutrient supply.^314^, ^315^, ^316^ In addition, it can also extend the lifespan of organoids by promoting vascularization, enabling the transport of nutrients and metabolic wastes. Coculture of organoid cells with endothelial cells or their progenitors can promote vascularization during organoid formation. 3D‐vascularized liver organoids, which were cultured with organoid chip technology, significantly improved intercellular interactions and metabolic activity.^317^ Besides, bioengineering methods, including sacrificial molds,^318^, ^319^ laser ablation,^320^, ^321^ and bioprinting,^322^, ^323^ can also produce vessel‐like structures. On the other hand, the cells used to generate organoids that are mainly epithelium‐originated cells, including normal or tumor cells. The microenvironment in which cells in the body are located comprises interstitial cells, immune cells, and matrix components. The current organoid field fails to reflect the complexity and cell–cell interactions of the native tissue. Unique in vivo environmental factors that encourage organoid maturation may be absent from in vitro conditions. Organoids tend to develop a more mature phenotype when implanted in vivo, indicating that exposure to the in vivo microenvironment will increase the development and maturity of organoids. When constructing hepatobiliary organoids, it is better to coculture them with mesenchymal cells. Because they can better promote organoid maturation,^196^ and can provide certain paracrine signals to induce cell differentiation and the formation of the 3D spatial structure of the corresponding organ.^324^

Finally, the ECM, which plays a role in supporting the 3D structure and providing ECM signals, impacts the development of organoids. The most commonly used is Matrigel, an ECM protein mixture secreted by mouse Englebreth‐Holm‐Swarm sarcoma cells, which contains many growth factors, such as insulin‐like growth factor 1 and EGF. However, different batches or the same batch of Matrigel might have inconsistent biochemical properties. As a nonhuman‐derived material, Matrigel might introduce viral or xenogenic contaminants to induce immune responses, which can interfere with organoid behavior and constrain the capacity to induce organoid morphogenesis.^325^ Matrigel leads to slower penetration of exogenous substances, which is not conducive to transfection and drug screening. Besides, Matrigel cannot yet fully mimic the true tumor stroma because tumor cells have features of anchorage‐independent growth. The above shortcomings may restrict its clinical applications. Therefore, significant efforts have been devoted to developing artificially synthesized ECM or hydrogels for organoid culture, which are chemically defined with clear components, and their chemical and physical features can be adjusted on the basis of different requirements.^326^, ^327^, ^328^, ^329^, ^330^, ^331^ The advantages of these novel hydrogels have been demonstrated. A nanofibrillar hydrogel proposed by Prince et al.^332^ could support the initiation and growth of breast tumoroids and protect the PDOs from murine contamination. Decellularized ECM hydrogels derived from various species have been employed in 3D culture, because they can promote proliferation and differentiation and enhance the attachment ability of organoid cells.^333^, ^334^, ^335^, ^336^ Liu's group recently developed a hydrogel based on the decellularized uvea with excellent cytocompatibility with iPSC‐derived retinal organoids. Animal experiment results indicated that after transplantation, retinal organoids cultured in this hydrogel showed an improved survival rate and were able to restore the vision of retinal degeneration in rats.^337^

Organoids provide unprecedented opportunities to study the development, physiology, and diseases of humans, comprising novel preclinical models with promising application prospects. Organoid models conserve the histological and gene expression properties of native tissue in vivo, and their high similarity to native tissue makes them a valuable addition to cell lines and animal models, which have a high clinical and scientific value. However, the organoid technique is still in the initial stage and has some shortcomings. Researchers should focus on combining this technique with other advanced technologies and models to further improve the accuracy of research in the future.

## AUTHOR CONTRIBUTION

Siqi Yang and Haijie Hu contributed equally to the manuscript and were the first coauthors. Siqi Yang and Haijie Hu contributed to data acquisition and drafted the manuscript. Yafei Hu, Yushi Dai, Ruiqi Zou, Tiantian Wang, Tianrun Lv and Hengchung Kung contributed to data acquisition. Fuyu Li, Jun Yu, and Haijie Hu contributed to the study design and revision of the manuscript. All authors read and approved the final manuscript.

## CONFLICT OF INTEREST STATEMENT

The authors have no conflicts of interest to disclose.

## ETHICS STATEMENT

Not applicable.

## Data Availability

Not applicable.
